# Equilibrium sampling of HOCs in sediments and suspended particulate matter of the Elbe River

**DOI:** 10.1186/s12302-018-0159-8

**Published:** 2018-08-02

**Authors:** Nora Claire Niehus, Sabine Schäfer, Christel Möhlenkamp, Gesine Witt

**Affiliations:** 1 Hamburg University of Applied Sciences, Ulmenliet 20, 21033 Hamburg, Germany; 2 German Federal Institute of Hydrology, Am Mainzer Tor 1, 56068 Koblenz, Germany

**Keywords:** Passive sampling, SPME, *C*_free_, HOCs, SPM, Sediment, Elbe River

## Abstract

**Background:**

Chemical quality of sediment and suspended particulate matter (SPM) is usually assessed by total chemical concentrations (*C*_total_). However, the freely dissolved concentration (*C*_free_) is the ecologically more relevant parameter for bioavailability, diffusion and bioaccumulation. In recent studies, equilibrium sampling has been applied to determine *C*_free_ of hydrophobic organic contaminants (HOCs) in the sediment pore water, whereas such data are missing for SPM. We applied solid-phase micro-extraction to measure and compare *C*_free_ of PAHs and PCBs in pore water of sediments and SPM sampled along the German part of the river Elbe. Moreover, site-specific distribution ratios were evaluated and *C*_bio,lipid_ was predicted using *C*_free_.

**Results:**

*C*_free_ of PAHs remained largely constant while *C*_free_ of PCBs varied along the Elbe River. The highest *C*_total_ of PCBs and PAHs were found at Prossen (km 13) and Meißen (km 96). PCB *C*_total_ even exceeded the environmental quality standard for sediment and SPM in Prossen. Site-specific distribution ratios (*K*_D_) revealed a stronger sorption for PAHs compared to PCBs, indicating a higher availability of PCBs. Equilibrium partitioning concentrations in lipids (*C*_lip↔sed_) showed a high correlation with actually measured lipid-normalised concentrations (*C*_bio,lipid_) in bream. This indicates that PCB bioaccumulation in this benthic fish species is closely linked to the sediment contamination.

**Conclusions:**

In rivers, SPM functions as a transportation vehicle for HOCs along the stream until it eventually deposits to the sediment. This study demonstrates that due to weaker sorption of PAHs and PCBs to the SPM this matrix poses a higher risk to the aquatic environment compared to the sediment. The prediction of *C*_bio,lipid_ of PCBs was correct and shows that solid-phase micro-extraction is highly suited to predict lipid concentration, and thus a valuable tool for risk-assessment or sediment management.

**Electronic supplementary material:**

The online version of this article (10.1186/s12302-018-0159-8) contains supplementary material, which is available to authorized users.

## Background

In terms of the assessment of sediment or suspended particulate matter (SPM), the total concentration (*C*_total_) is not the deciding factor when describing ecotoxicological impacts. To assess accessibility/bioavailability, diffusion or bioaccumulation, it is well known that the relevant parameter is the chemical activity (*a*). The chemical activity is the substance-specific potential for spontaneous physicochemical processes. *a* is not coupled to a phase such as the freely dissolved concentration (*C*_free_) is to the aqueous phase and thus diffusion or partition between different phases (e.g. water/sediment) can be described via chemical activity [1]. Since contaminants always partition from the high to low chemical activity the question whether a compartment is a sink or a source can be answered using this parameter. The chemical activity is proportional to *C*_free_:

1 $$a = C_{\text{free}} /S_{\text{L}}$$

where *S*_L_ is the subcooled liquid solubility of a sparingly soluble compound in water. Equilibrium sampling is a rapid method to measure *C*_free_. The principle is based on a polydimethylsiloxane (PDMS)-coated glass fibre that is equilibrated in the sediment or SPM sample and then directly measured using GC–MS [2]. The analyte concentration in the PDMS (*C*_PDMS_) is then converted into *C*_free_ using analyte-specific polymer/water partitioning coefficients (*K*_PDMS:water_):

2 $$C_{\text{free}} = \, C_{\text{PDMS}} /K_{\text{PDMS:water}} .$$

Another parameter that completes the picture of bioavailability and accessibility is the site-specific distribution ratio (*K*_D_). *K*_D_ is the ratio of *C*_total_ and *C*_free_ and states the sorption strength of a contaminant to the sediment or SPM. To assess bioaccumulation, recent studies used equilibrium partitioning concentrations in lipids (*C*_lip↔sed_) for PCBs [3, 4]. These were obtained from equilibrium sampling in sediment and showed a high correlation with actual measured lipid-normalised concentrations in biota.

In the present study, the river Elbe was selected as study area. The Elbe River rises in the Czech Republic and flows through Germany where it enters the North Sea at Cuxhaven. In total, the length of the Elbe River is 1094 km. Although a majority of the Elbe flows through Germany, the main contribution to the polychlorinated biphenyl (PCBs) contamination of the Elbe originates from the Czech Republic [5]. Another big issue is the Hamburg Harbour and the sediment management. Due to increasing draught of ships and sedimentation, Elbe sediments are regularly dredged to maintain the economic performance of the Hamburg Harbour. Moreover, as one of the largest rivers in Europe, the Elbe significantly contributes to the pollution load of the southern North Sea. There have been several monitoring programmes, however, only *C*_total_ was monitored. In several studies equilibrium sampling using SPME fibres has been successfully applied to determine *C*_free_ of HOCs in the sediment pore water [2, 6], whereas such data are missing for SPM. As one of the important pathways for HOCs to enter the aquatic environment, SPM plays major role to understand the partition in the riverine system.

In the present study, we applied SPME to measure *C*_free_ of PAHs and PCBs in the pore water associated with the sediment and SPM of the German part of the river Elbe. This study aims to

(i) Provide the first dataset of *C*_free_ of HOCs in SPM and compare these data with sediments.
(ii) Predict the internal PCB concentrations in biota using equilibrium sampling in sediment.
(iii) Show the downstream pattern of *C*_total_ and *C*_free_ of the sediment/SPM pore water.
(iv) Identify site-specific distribution ratios (*K*_D_) and the influence of total organic carbon (TOC) and soot.

## Methods

### Sampling

**Fig. 1:**
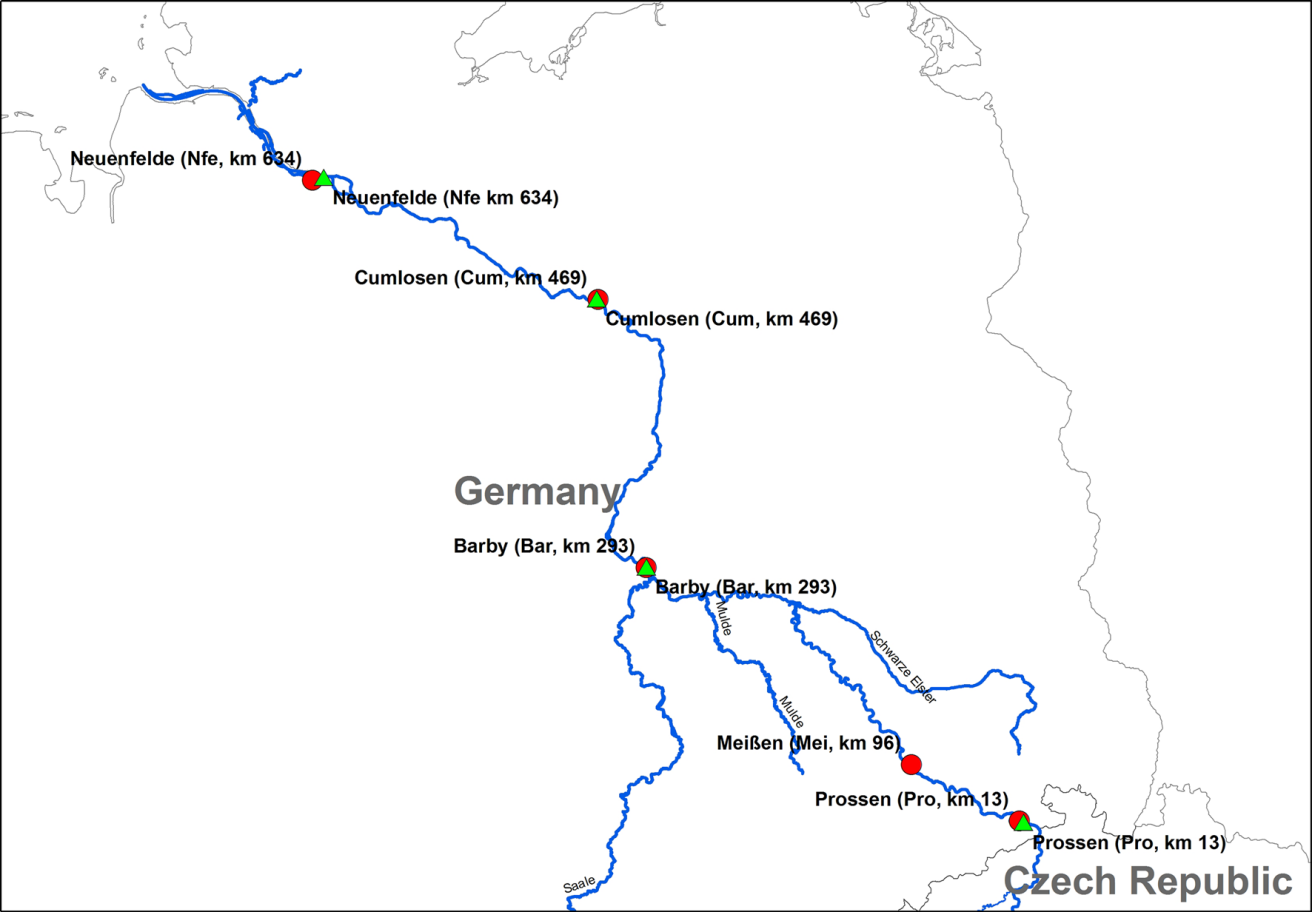
Sampling stations along the Elbe River. Sediment (red dots) and SPM (green triangles) samples were collected at Neuenfelde (Nfe, 634 river km), Cumlosen Barby (Bar, 293 river km), Meißen (Mei, 96 river km) and Prossen (Pro, 13 river km)

Sediment from Neuenfelde (Nfe, 634 km) was collected in September 2014 using a van Veen-type grab sampler (top layer 0–20 cm), homogenised, filled into pre-cleaned aluminium boxes and stored at − 20 °C until analysis. Sediments from Cumlosen (Cum, 469 km), Barby (Bar, 293 km), Meißen (Mei, 96 km) and Prossen (Pro, 13 km) were collected in July 2014. All sampling stations are shown in Fig. 1. Kilometre zero of the sampling points along the River Elbe begins at the German–Czech Border. The sediments were collected with a van Veen-type grab sampler (top layer 0–20 cm), homogenised but stored in plastic buckets at + 4 °C. From these samples, approximately 500 g aliquots were transferred into pre-cleaned aluminium boxes, transported to the HAW and stored at − 20 °C until analysis.

SPM bulk samples were collected in September 2014 and treated according to Ricking et al. [7]. Briefly, sedimentation boxes are permanently installed in federal monitoring stations with a flow rate of 8–10 L min^−1^. For sampling, supernatant water was removed and the remaining SPM was sieved (2 mm), homogenised and subsequently frozen on site. Frozen samples were stored in stainless steel containers and transported to the depot of the German Environment Specimen Bank (ESB) in a nitrogen vapour freezer. SPM samples were filled in amber glass bottles and stored at + 4° C until analysis. The geographical positions of the sampling stations are listed in the Additional file 1: Table S2.

### Exhaustive extraction of sediments and SPM (*C*_total_)

#### Sediments

Total concentrations of seven PCBs (PCB 28, 52, 101, 118, 138, 153, 180) and 16 PAHs (naphthalene, acenaphthylene, acenaphthene, fluorene, phenanthrene, anthracene, fluoranthene, pyrene, benzo[a]anthracene, chrysene, benzo[b]fluoranthene, benzo[k]fluoranthene, benzo[a]pyrene, indeno[cd-1,2,3]pyrene, dibenzo[a]anthracene and benzo[ghi]perylene) were determined in sediment samples. Freeze-dried sediments were sieved (< 2 mm) and ground. Approximately 5 g freeze-dried sediment of the original homogenised grab sample was extracted in a pressurised liquid extractor (ASE 200, Dionex, Idstein, Germany). Reduced extracts were cleaned with a copper–aluminium oxide column and subsequently fractionated with gel permeation chromatography using a ShodexTM CLNpakPAE 800 AC 8.0 × 300 mm column (Shodex Buisness, Showa Denko Europe GmbH, Munich, Germany) and acetone as eluent. The collected fractions were analysed by gas chromatography with triple quadrupole mass spectrometric detection (Chromtech Evolution Systems, Chromtech GmbH, Idstein, Germany).

#### SPM

Total concentrations of seven PCBs (PCB 28, 52, 101, 118, 138, 153, 180) and 16 PAHs (naphthalene, acenaphthylene, acenaphthene, fluoren, phenanthrene, anthracene, fluoranthene, pyrene, benzo[a]anthracene, chrysene, benzo[b]fluoranthene, benzo[k]fluoranthene, benzo[a]pyrene, indeno[cd-1,2,3]pyrene, dibenzo[a]anthracene and benzo[ghi]perylene) were determined in SPM samples. For this, SPM samples were freeze-dried using a Christ freeze drier (Alpha 1–4 LD plus). The extraction was performed according to Witt et al. [2] with minor modifications. Briefly, 3–4 g of freeze-dried samples was extracted using an accelerated solvent extractor (ASE 2000, DIONEX). The samples were filled in ASE cartridges with an internal standard (PAH Mix-9 deuterated, Dr. Ehrenstorfer; PCB Mixture EC-4058, Cambridge Isotope Laboratories) and extracted with 60 mL acetone–hexane (v/v: 40/60) at 140 bar and 100 °C. The concentrated extracts were fractionated by HPLC with a silica gel column (MERCK, LiChrospher Si 100-5). Elemental sulphur was removed with 0.5 g activated copper. The copper was activated with HCl and then cleaned with methanol, acetone and *n*-hexane. The extracts were then concentrated to 500 µL and subsequently measured using a GC–MS.

### Total organic carbon (TOC)

For the analysis of TOC, 100–700 mg of freeze-dried sediment/SPM was acidified with 1 mL hydrochloric acid (1 M) for 3–4 h and subsequently measured in a TOC analyzer (Eltra Helios, Eltra, Neuss, Germany). CNS content was measured with a CNS analyzer (Vario Macro, Elementar, Hanau, Germany) by weighing 20–120 mg of freeze-dried sample, depending on the TOC content, and adding 120% tungsten (VI) oxide for quantification of sulphur. In the CNS analyzer, the sediment was quantitatively decomposed into gaseous compounds by high temperatures. The sample was cleaned, separated in its components and elements were quantified by respective detectors.

### Equilibrium sampling (*C*_free_)

*C*_free_ of seven PCBs (PCB 28, 52, 101, 118, 138, 153, 180) and 12 PAHs (phenanthrene, anthracene, fluoranthene, pyrene, benzo[a]anthracene, chrysene, benzo[b]fluoranthene, benzo[k]fluoranthene, benzo[a]pyrene, indeno[cd-1,2,3]pyrene, dibenzo[a]anthracene and benzo[ghi]perylene) were determined in pore water of sediment and SPM samples. Equilibrium sampling of HOCs in sediment and SPM was performed as described previously [2, 6] with minor modifications. Briefly, 7–10 g of thawed and homogenised sediments or SPM was filled in 12-mL round-bottom glass vials with polytetrafluoroethylene (PTFE) septum caps. Three 10-cm SPME fibres (Fiberguide Industries Inc.) with a 10-µm PDMS coating were placed in each vial. All vials were agitated on an overhead shaker in darkness at 20 °C for 14 days. SPME fibres were removed and immediately cleaned with Milli-Q water, dried with lint-free tissue and stored at − 20 °C in pre-cleaned aluminium foil. The HOCs in the fibres were analysed using thermal desorption in a GC–MS system. After the measurement, analyte concentrations in PDMS (*C*_PDMS_) were calculated based on the measured length and the specific volume of the fibre (0.0877 µL cm^−1^). *C*_free_ was then calculated with Eq. 2 using PDMS to water partitioning coefficients (*K*_PDMS:water_) obtained from Witt et al. [8].

### Site-specific distribution ratios (*K*_D_)

Partitioning of HOCs between the sediment or SPM (*C*_total_) and the pore water or the water column (*C*_free_) is an indicator for the sorption strength of a specific compound and depends on the composition of the sediment or SPM (e.g. TOC, soot) and its pore water or surrounding water (e.g. DOC) as well as external factors such as temperature. Thus, *K*_D_ values are highly specific values for each sample and analyte and indicate the availability of HOCs. The site-specific distribution ratios were calculated with the following equation:

$$K_{D} = \frac{{C_{\text{total}} }}{{C_{\text{free}} }}.$$

### Bioaccumulation

PCB concentrations in muscle tissue from *Abramis brama* (*C*_bio_) in ng kg^−1^ wet weight (ww) and fat contents (%) were obtained from the ESB. These fish samples were collected in 2014 during a monitoring and at the same sampling stations of this study (Additional file 1: Table S1). Since PCBs mainly accumulate in lipid, *C*_bio_ was normalised to the lipid content (*C*_bio,lipid_) in µg kg^−1^ lipid.

Equilibrium partitioning concentrations in fish lipid (*C*_lip↔sed_) were obtained by multiplying *C*_PDMS_ with analyte-specific partitioning coefficients between lipid and PDMS (*K*_lip:PDMS_) [4, 9, 10]:

$$C_{{{\text{lip}} \leftrightarrow {\text{sed}}}} = C_{\text{PDMS}} *K_{\text{lip:PDMS}} .$$

And *K*_lip:PDMS_ was obtained by

$$K_{\text{lip:PDMS}} = \frac{{K_{\text{lip:SSP}} }}{{K_{\text{PDMS:SSP}} }}.$$

Partitioning ratios between lipid and the silicone SSP-M823 (Speciality Silicone Products Inc.) (*K*_lip:SSP_) were obtained from Jahnke et al. [9] and ratios between PDMS fibres and SSP (*K*_PDMS:SSP_) were published by Gilbert et al. [11].

## Results

### Total concentrations (*C*_total_) in sediment and SPM

**Fig. 2:**
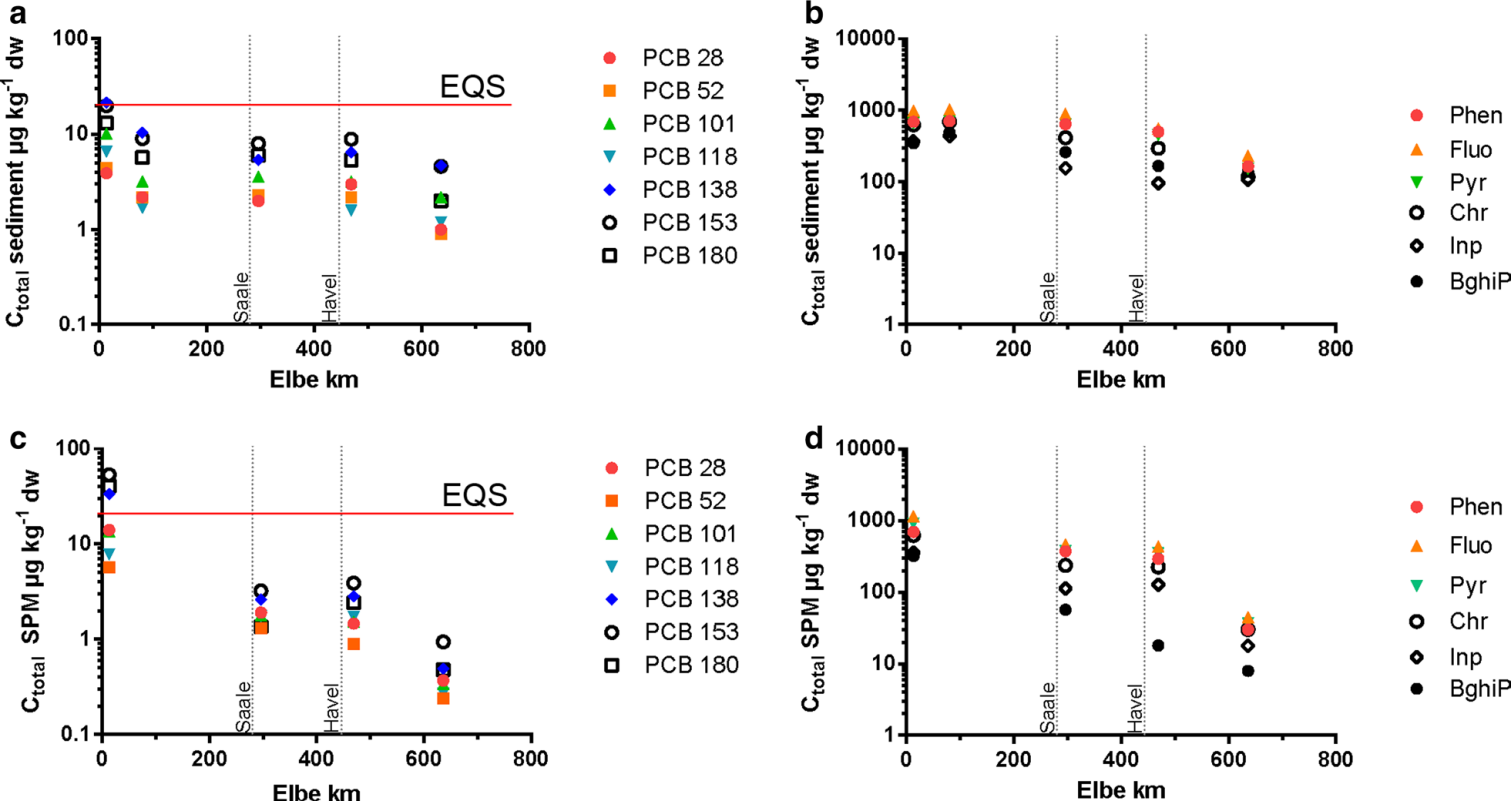
*C*_total_ (µg kg^−1^ dry weight) of PCBs (**a**, **c**) and selected PAHs (**b**, **d**) in sediment and SPM along the German part of the River Elbe. The red line marks the environmental quality standard (EQS) of 20 µg kg^−1^ for single PCBs in sediment and SPM (German Surface Water Regulation). Data points are single measurements

*C*_total_ of sediment and SPM samples is plotted over river kilometres in Fig. 2. In sediment samples, *C*_total_ ranged from 1677 to 6359 μg kg^−1^ for the sum of 11 PAHs^1^ and 17 to 80 μg kg^−1^ for the sum of the seven measured PCBs. In SPM, *C*_total_ concentrations ranged from 292 to 6580 μg kg^−1^ for the sum of 11 PAHs and 7–169 μg kg^−1^ for the sum of seven PCBs.

In both matrices, the highest concentrations of PCBs and PAHs were found at Prossen (km 13) and Meißen (km 96) with a decreasing trend downstream the River Elbe. Regarding the sums of PAHs and PCBs, there was no substantial difference in contaminant levels between sediment and SPM samples, except for Prossen which had elevated PCB concentrations in SPM of 169 μg kg^−1^ which is twice the *C*_total_ of the sediment with 79 µg kg^−1^. At this station, the environmental quality standard of 20 μg kg^−1^ (for single PCBs in sediment and SPM, German Surface Water Regulation [12]) is exceeded for PCB 138 and 153 in the sediment and for PCB 138, 153 and 180 in the SPM. Single concentrations of *C*_total_ of sediment and SPM samples are available in the Additional file 1: Tables S3–S6.

### Freely dissolved concentrations (*C*_free_) in sediment and SPM

**Fig. 3:**
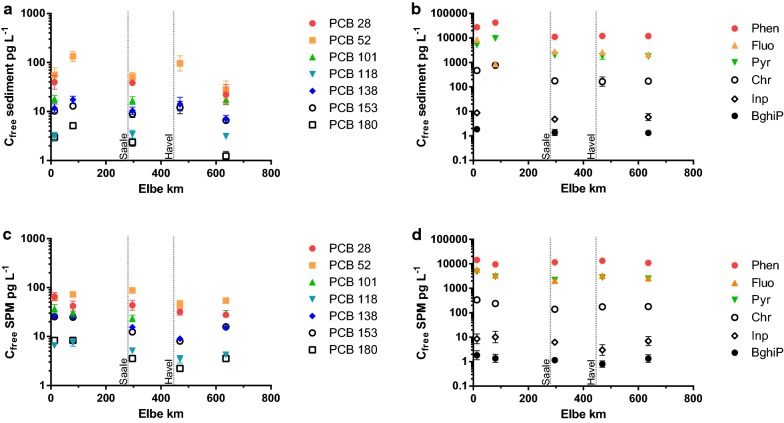
*C*_free_ (pg L^−1^) of PCBs (**a**, **c**) and selected PAHs (**b**, **d**) in sediment and SPM samples along the German part of the River Elbe. Data points are mean values and the error bars represent the standard deviations. *C*_free_ sediment *n* = 3 (Meißen km 80, *C*_free_ *n* = 2). *C*_free_ SPM *n* = 3 (Barby km *C*_free_ *n* = 2)

*C*_free_ of single PAHs in sediments and SPM were similar and largely remained constant along the River Elbe (Fig. 3). For single PCBs, *C*_free_ varied along the Elbe and particularly in sediment samples from Meißen and Cumlosen. In sediment samples, *C*_free_ ranged from 15 to 60 ng L^−1^ for the sum of 11 PAHs (see footnote 1) and from 56 to 233 pg L^−1^ for the sum of the seven measured PCBs. *C*_free_ in SPM ranged from 22 to 33 ng L^−1^ for the sum of 11 PAHs and from 178 to 281 pg L^−1^ for the sum of seven PCBs. For PCBs and PAHs, the highest *C*_free_ in sediment and SPM was found in Meißen (Additional file 1: Tables S7–S10).

### Sorption of HOCs

**Fig. 4:**
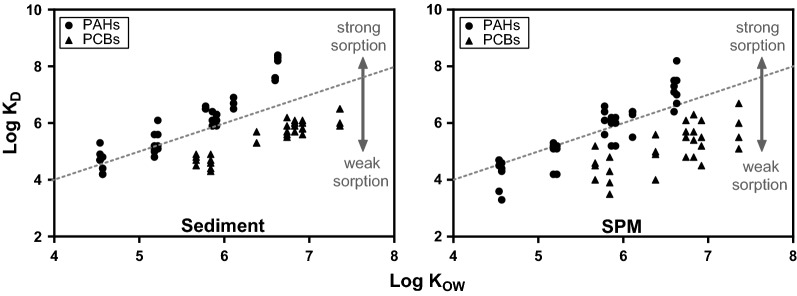
Site-specific distribution ratios (*K*_D_) values plotted over Log *K*_OW_ of PAHs and PCBs for sediment and SPM samples. *K*_D_ values were calculated for five sediment stations with *C*_free_ *n* = 3, *C_free_ *n* = 1; for SPM *C*_free_ *n* = 4 and for Prossen *n* = 6. *C*_total_ of sediment and SPM are single measurements

*K*_D_ values were calculated by dividing *C*_total_ by *C*_free_ and plotted over log *K*_OW_ values (Fig. 4). Distribution coefficients of PAHs and PCBs in sediments among all stations differed only marginally. In sediment, *K*_D_ values for PAHs and PCBs are clearly separated. *K*_D_ values for PAHs were higher and mostly above the 1:1 ratio, whilst *K*_D_ values for PCBs are well below that ratio. This demonstrates a stronger sorption of PAHs to the sediment compared to PCBs. In SPM, *K*_D_ values for PAHs and PCBs among stations differed substantially and the separation of PAH and PCB was not visible. For SPM, only slight differences in the sorption strength between PAHs and PCBs were observed.

### Influence of TOC and soot

**Fig. 5:**
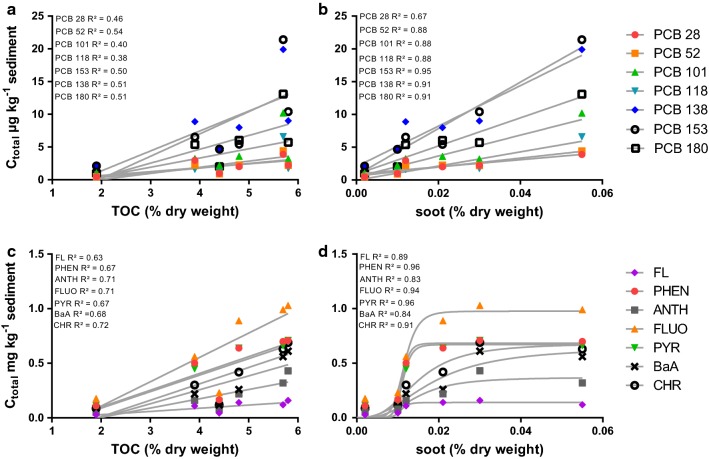
Total concentrations (*C*_total_, *n* = 1) in Elbe sediments correlated with TOC or soot content for PCBs (**a**, **b**) and PAHs (**c**, **d**). *R*^2^ values were obtained with a linear regression for **a**, **b** and **c** and for **d** with a specific-binding with Hill-slope equation

The different sorption strengths raised the question as to what influence does the TOC or soot content have in these particular sediments. To clarify this question, *C*_total_ values of PAHs and PCBs were correlated with the TOC and soot contents in percent dry weight (dw) of Elbe sediments (Fig. 5). The linear correlation demonstrates that the PCBs in the Elbe sediments clearly prefer soot (*R*^2^ 0.67–0.95) over TOC (*R*^2^ 0.38–0.54). For PAHs, the linear relationship with TOC is visible (*R*^2^ 0.63–0.72), only the sampling station Neuenfelde with a TOC content of 4.3% dw affects this relationship. However, the correlation with soot revealed a saturation curve with higher *R*^2^ values between 0.83 and 0.96.

### Linking *C*_free_ and *C*_biota_

Several studies have shown a close link between sediment contamination and bioaccumulation of PCBs in different benthic organisms [3, 4, 13, 14]. The authors applied coated jars to measure the concentration in the PDMS (*C*_PDMS_) with sediment samples und used this to predict the concentration in the lipid of fish (*C*_lip_) using partitioning coefficients between silicone and lipid.

**Fig. 6:**
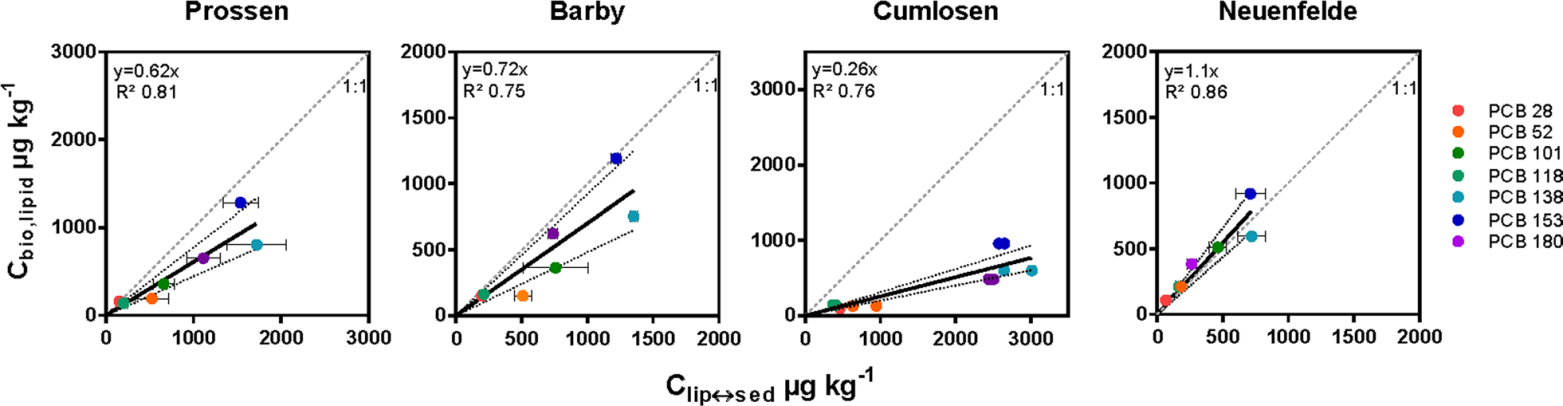
Lipid normalised concentrations of PCBs in muscle tissue of *Abramis brama* (*C*_bio,lipid_ in µg kg^−1^ lipid) plotted over equilibrium concentrations (*C*_lip↔sed_ in µg kg^−1^ lipid) for the stations Prossen (km 13), Barby (km 296), Cumlosen (km 470) and Neuenfelde (km 636). The limit of detection (LOD) was 3.3 µg kg^−1^ wet weight and for Barby PCB 28 and PCB 52 and for Cumlosen PCB 28 were exactly on this LOD. *C*_lip↔sed_ values are mean values (*n* = 3) and error bars are standard deviations. For Cumlosen only two replicates are available of which both are presented. *C*_bio,lipid_ values are single measurements. Dashed lines represent 1:1 relationship and dotted lines are confidence intervals of the linear regression forced through the origin

In Fig. 6, the predicted concentration in lipid (*C*_lip↔sed_) was plotted against the lipid-normalised PCB concentrations in muscle tissue of common bream (*Abramis brama*). Linear regressions yielded high *R*^2^ values for Prossen, Barby, Cumlosen and Neuenfelde of 0.81, 0.75, 0.76 and 0.86, demonstrating the close link between sediment contamination and fish bioaccumulation and thus the transfer of PCBs from the sediment to biota. In Prossen, Barby, and Cumlosen, the prediction was at or below the equilibrium, whilst at Neuenfelde the actual contamination in common bream was slightly higher than the predicted contamination.

## Discussion

### Total concentrations (*C*_total_) in sediment and SPM

*C*_total_ of PCBs and PAHs in sediments and SPM was in the same order of magnitude as results from other studies on the Elbe [15–18].

The exceedance of the environmental quality standard in sediment and SPM at Prossen indicates a recent contamination near the German–Czech border. These findings agree with a monitoring programme by the RBC Elbe that revealed an exceedance of the EQS (20 µg kg^−1^ in sediment and SPM, WFD) since 2012 with an increasing trend. This PCB contamination mainly consists of higher chlorinated PCBs, which are presumed to originate from a Škoda plant near Prossen.

### Freely dissolved concentrations (*C*_free_) in sediment and SPM

*C*_free_ of PCBs and PAHs determined in the present study in sediments from the Elbe was in the same order of magnitude compared to sediment data from the Elbe [2, 4]. For PCBs and PAHs, the highest *C*_free_ in sediment and SPM was found in Meißen, while the highest *C*_total_ was found in Prossen. This demonstrates that a high *C*_total_ does not necessarily result in high *C*_free_ values. This indicates a stronger sorption of HOCs to the sediment in Prossen which is supported by high site-specific distribution ratios determined in the present study (Fig. 3). *C*_free_ of PCBs is further comparable to levels in sediments from the Baltic Sea [6]. *C*_free_ of PAHs was further comparable to sediment data from the Oslo Harbor [19], whereas they were six times higher compared to data from the Baltic Sea [6]. Consequently, the PCB and PAH contamination level in the river Elbe can be characterised as moderately elevated.

### Sorption of HOCs

Up to now, this is the first study that used SPME to measure *C*_free_ of HOCs in SPM. Although it is rather unlikely, that SPM and the surrounding water will establish an equilibrium under realistic conditions, the ratio of *C*_total_ and *C*_free_ (*K*_D_) helps to understand the partitioning of HOCs between SPM and water and thus the sorptive behaviour to SPM. For SPM, Fig. 4 shows a weaker sorption and generally more scattered *K*_D_ values compared to the sediment. For the sediment, Fig. 4 demonstrates stronger sorption of PAHs compared to PCBs. These different sorptive behaviours of PAHs and PCBs to the sediment are in agreement with other studies Bucheli and Gustafsson [20] and Koelmans et al. [21]. Jonker and Koelmans [22] suggested that this results from different chemical structures of PAHs and PCBs. They concluded that pore sorption in soot is the main factor for PCBs and PAHs. Additionally, PAHs are able to from non-covalent interactions because of their planar structure. This indicates that when there are more possibilities for PAHs to develop a bond, the sorption of PAHs to the sediment is stronger. We suggest that these different sorption capacities in both matrices result from ageing processes during and after sedimentation of SPM to the sediment.

### Influence of TOC and soot

The findings of the present study agree with those of Koelmans et al. [21], who concluded a saturation of the soot content. When the soot content is saturated, HOCs would then bind to the TOC content. Finally, we demonstrated that PCBs and PAHs prefer binding to soot over TOC and thus that soot-like material is highly suited for remediation applications in aquatic systems.

### Linking *C*_free_ and *C*_biota_

For all sampling sites, we found a high correlation of *C*_bio,lipid_ and *C*_lip↔sed_ indicating that bioaccumulation of PCBs in this benthic fish species is linked to the sediment contamination and moreover to the bioavailable fraction (*C*_free_) rather than *C*_total_. This was previously demonstrated by Schäfer et al. [4] for Elbe sediments. However, in the present study, *C*_lip↔sed_ in Neuenfelde was slightly higher compared to the lipid-normalised PCB concentrations in bream. The equilibrium partitioning theory assumes that the equilibrium cannot be exceeded but if so, it could indicate biomagnification [23]. However, since *C*_lip↔sed_ is normalised to a generic lipid reflecting an ideal organism results might slightly differ. Previous studies showed that the majority of predictions of *C*_bio,lipid_ were below or at equilibrium and did not markedly exceed the equilibrium [4, 10, 3, 13]. Moreover, considering that the *C*_free_ and *C*_biota_ were investigated independently, these findings demonstrate that passive sampling is in general suitable to predict the internal contamination of organisms living close to or in the sediment.

## Conclusion

In this study, we applied equilibrium sampling to compare PCB and PAH contamination of SPM and sediment from the German part of the River Elbe. We demonstrate that *C*_total_ of PCBs and PAHs clearly showed a decreasing trend downstream the River Elbe in SPM and sediment whereby the decrease was stronger in the SPM matrix. *C*_free_ remained largely unchanged for PAHs and varied for PCBs. This again underlines that *C*_total_ does not reflect the actual exposure. The highest *C*_total_ was measured in Prossen and exceeded the environmental quality standard (EQS of the German Surface Water Regulation) for *C*_total_ of PCBs in sediments and SPM. We further observed similar *C*_total_ in sediment and SPM (except for Prossen). However, in SPM HOCs were better available due to a weaker sorption, which indicates that SPM poses a higher risk to the aquatic environment compared to the sediment. The correlation of *C*_total_ to the TOC and soot content revealed a strong preference for soot and we further demonstrated that the soot content in Elbe sediments was saturated with PAHs. Moreover, the present study confirms the close link between sediment contamination and bioaccumulation, which further demonstrates that equilibrium sampling is highly suited to predict lipid concentration, and thus a valuable tool for risk-assessment or sediment management. In summary, parameters derived from equilibrium sampling (*C*_free_/chemical activity, *K*_D_, *C*_lip↔sed_) are valuable for today’s sediment assessment and should be a part of the sediment management.

## Additional file

**Additional file 1: Table S1.** Biometric data and information on the tissue treatment of the sampled fish. **Table S2.** Sampling stations in the German part of the River Elbe including the station name, river km, matrix (sediment or suspended particulate matter (SPM)), the geographical position (latitude, longitude) and the sampler. Sediment samples were collected in July and SPM in September 2014. **Table S3.** Ctotal of seven PCBs in sediment samples from the River Elbe in µg kg^−1^ (dw). **Table S4.** C_total_ of PAHs in sediment samples from the River Elbe in µg kg^−1^ (dw). **Table S5.** Ctotal of seven PCBs in SPM samples from the River Elbe in µg kg^−1^ (dw). **Table S6.** Ctotal of PAHs in SPM samples from the River Elbe in µg kg^−1^ (dw). **Table S7.** Cfree of PCBs in sediment samples from the River Elbe in pg L^−1^. **Table S8.** C_free_ of PAHs in sediment samples from the River Elbe in pg L^−1^. **Table S9.** C_free_ of PCBs in SPM samples from the River Elbe in pg L^−1^. **Table S10.** C_free_ of PAHs in SPM samples from the River Elbe in pg L^−1^.

## Abbreviation

*C*_bio,lipid_: lipid-normalised concentration of PCBs in *Abramis brama*
*C*_free_: freely dissolved concentration in the pore water of the sediment or suspended particulate matter
*C*_lip↔sed_: lipid concentration calculated from a potential equilibrium between *C*_free_ of the sediment pore water and a generic lipid
*C*_PDMS_: concentration in the PDMS
*C*_total_: total concentration in the sediment or suspended particulate matter
ESB: German Environment Specimen Bank
EQS: environmental quality standard
HOCs: hydrophobic organic chemicals
*K*_D_: site-specific distribution ratio (*C*_total_/*C*_free_)
K_lip:PDMS_: partitioning coefficient between a generic lipid and PDMS
K_lip:SSP_: partitioning coefficient between a generic lipid and SSP-M823
K_PDMS:SSP_: partitioning coefficient between PDMS and SSP-M823
K_PDMS:water_: partitioning coefficient between PDMS and water
PAH: polycyclic aromatic hydrocarbons
PCBs: polychlorinated biphenyls
PDMS: polydimethylsiloxane
SPM: suspended particulate matter
SPME: solid-phase micro-extraction
TOC: total organic carbon
WFD: Water Framework Directive

## Footnotes

1 Phenanthrene, anthracene, fluoranthene, pyrene, benzo[a]anthracene, chrysene, benzo[b]fluoranthene, benzo[k]fluoranthene, benzo[a]pyrene, indeno[c,d-1,2,3]pyrene and benzo[ghi]perylene.
