# Supplementary material for: Equilibrium sampling of HOCs in sediments and suspended particulate matter of the Elbe River
Source: Environ Sci Eur. 2018 Aug 2;30(1):28. doi: 10.1186/s12302-018-0159-8 (PMC6097018; doi:10.1186/s12302-018-0159-8)
Supplement: Supplementary file 1 — Additional file 1: Table S1. Biometric data and information on the tissue treatment of the sampled fish. Table S2. Sampling stations in the German part of the River Elbe including the station name, river km, matrix (sediment or suspended particulate matter (SPM)), the geographical position (latitude, longitude) and the sampler. Sediment samples were collected in July and SPM in September 2014. Table S3. Ctotal of seven PCBs in sediment samples from the River Elbe in µg kg−1 (dw). Table S4. Ctotal of PAHs in sediment samples from the River Elbe in µg kg−1 (dw). Table S5. Ctotal of seven PCBs in SPM samples from the River Elbe in µg kg−1 (dw). Table S6. Ctotal of PAHs in SPM samples from the River Elbe in µg kg−1 (dw). Table S7. Cfree of PCBs in sediment samples from the River Elbe in pg L−1. Table S8. Cfree of PAHs in sediment samples from the River Elbe in pg L−1. Table S9. Cfree of PCBs in SPM samples from the River Elbe in pg L−1. Table S10. Cfree of PAHs in SPM samples from the River Elbe in pg L−1. [file 12302_2018_159_MOESM1_ESM.docx]

Additional file 1

Equilibrium sampling of HOCs in sediments and suspended particulate matter of the Elbe River

Nora Claire Niehus^1^, Sabine Schäfer^2^, Christel Möhlenkamp^2^, Gesine Witt^1^

^1^ Hamburg University of Applied Sciences, Ulmenliet 20, 21033 Hamburg

² German Federal Institute of Hydrology, Am Mainzer Tor 1, 56068 Koblenz

Email:

Nora Niehus: Nora.Niehus@rwth-aachen.de

Sabine Schäfer: Sabine.Schaefer@bafg.de

Christel Möhlenkamp: Moehlenkamp@bafg.de

Gesine Witt: gesine.witt@haw-hamburg.de

Corresponding author: Nora Claire Niehus

**Text S1:** Fish sampling by the ESB

Sampling of common bream (Abramis brama) is performed yearly according to the ESB standard operating procedure (UBA 2012). In general, female and male individuals of A. brama are sampled after spawning period in August and September. The age of the fish is determined in the laboratory on the basis of scales and operculum. At least 20 individuals ( 8 to 12 years) are sampled at each location and their biometric data are recorded. The fish are sectioned and muscle tissue without skin are sampled. After pooling and homogenization, samples are stored at temperatures below -150°C for retrospective monitoring.

**Table S1.** Biometric data and information on the tissue treatment of the sampled fish.

| **Station** | **Weight, total (g)** | **Age (years)** | **Length (cm)** | **Weight, muscle (g)** | **Extraction method** | **Analysing method** |
| --- | --- | --- | --- | --- | --- | --- |
| Prossen (13 km) | 1414.6  ± 239.01 | 10.8  ± 3.97 | 50.45  ± 2.97 | 218.44  ± 36.04 | Soxhlet extraction (Hexane/  Dichloromethane) | GC/HRMS |
| Barby (296 km) | 1209.6  ± 346.45 | 10.5  ± 3.74 | 48.44  ± 4.81 | 171.91  ± 51.66 | Soxhlet extraction (Hexane/  Dichloromethane) | GC/HRMS |
| Cumlosen (470 km) | 1326.95  ± 154.56 | 7.7  ± 2.43 | 50.425  ± 2.18 | 194.69  ± 30.92 | Soxhlet extraction (Hexane/  Dichloromethane) | GC/HRMS |
| Blankenese (634 km) | 1494.5  ± 366.03 | 7.35  ± 2.03 | 50.43  ± 4.14 | 223.09  ± 48.32 | Soxhlet extraction (Hexane/  Dichloromethane) | GC/HRMS |

More detailed information’s on the fish sampling and treatment is described by Fliedner et al. (2018)

This monitoring data is online available at: <https://umweltprobenbank.de/de/documents/investigations/results/biometrics?sampling_areas=10037+10035+10033+10031+10002&sampling_years=2014..2015&specimen_types=10001> (Last accessed 25.06.2018 11:22)

*Animal welfare*

The German Federal Environment Ministry and the German Environmental Agency commission environmental experts at the University of Trier with the collection of environmental samples for the environmental specimen bank. Required sampling permits are obtained from fisheries authorities, conservation authorities or third parties. The sampling is based on the German Animal Welfare Act. Sampling procedures are determined and documented in the standard operating procedures (SOP) of the Environmental Specimen Bank.

*SOP*

UBA German Environmental Specimen Bank. German Federal Environment Agency (Umweltbundesamt. UBA), 2012. Richtlinie zur Probenahme und Probenbearbeitung: Brassen (Abramis brama). Verfahrensrichtlinien für Probenahme, Transport, Lagerung und chemische Charakterisierung von Umwelt- und Humanproben. Stand: August 2012. V 2.0.2. available at <http://umweltprobenbank.de/upb_static/fck/download/SOP_Brassen.pdf>

**Table S.2:** Sampling stations in the German part of the River Elbe including the station name, river km, matrix (sediment or suspended particulate matter (SPM)), the geographical position (latitude, longitude) and the sampler. Sediment samples were collected in July and SPM in September 2014.

| Station (abbreviation) | Elbe River km | Matrix | Latitude | Longitude |
| --- | --- | --- | --- | --- |
| \| Prossen (Pro) \| \| --- \| \|  \| | 13 | Sediment | 50.92773 | 14.11618 |
| Meißen (Mei) | 96 | Sediment | 51.17329 | 13.47242 |
| \| Barby (Bar) \| \| --- \| | 293 | Sediment | 51.99070 | 11.88214 |
| Cumlosen (Cum) | 469 | Sediment | 53.03956 | 11.64175 |
| Neuenfelde (Nfe) | 634 | Sediment | 53.53016 | 9.80656 |
| \| Prossen (Pro) \| \| --- \| \|  \| | 13 | SPM | 50.92086 | 14.14036 |
| \| Barby (Bar) \| \| --- \| | 293 | SPM | 51.99343 | 11.88485 |
| Cumlosen (Cum) | 469 | SPM | 53.04227 | 11.63398 |
| Neuenfelde (Nfe) | 634 | SPM | 53.54011 | 9.88066 |

**Table S3:** C_total_ of seven PCBs in sediment samples from the River Elbe in µg kg^-1^ (dw).

| Station | TOC (% dry weight) | soot (% TOC) | unit | PCB 28 | PCB 52 | PCB 101 | PCB 118 | PCB 153 | PCB 138 | PCB 180 |
| --- | --- | --- | --- | --- | --- | --- | --- | --- | --- | --- |
| Prossen | 5.69 | 0.97 | µg/kg | 3.9 | 4.4 | 10.2 | 6.6 | 19.9 | 21.4 | 13.1 |
| Meißen | 5.79 | 0.52 | µg/kg | 2.2 | 2.2 | 3.2 | 1.7 | 9.0 | 10.4 | 5.7 |
| Barby | 4.78 | 0.44 | µg/kg | 2.0 | 2.3 | 3.6 | 2.2 | 8.0 | 5.4 | 6.0 |
| Cumlosen | 3.94 | 0.31 | µg/kg | 3.0 | 2.2 | 3.2 | 1.6 | 8.9 | 6.5 | 5.4 |
| Neuenfelde | 4.44 | 0.23 | µg/kg | 1.0 | 0.9 | 2.2 | 1.2 | 4.6 | 4.7 | 2.0 |

**Table S4:** C_total_ of PAHs in sediment samples from the River Elbe in µg kg^-1^ (dw).

| Station | TOC (% dry weight) | Soot  (% TOC) | NAP | ACY | ACE | FLU | PHE | ANT | FLA | PYR | BaA | CHR | BbF | BkF | BaP | InP | DBA | BghiP |
| --- | --- | --- | --- | --- | --- | --- | --- | --- | --- | --- | --- | --- | --- | --- | --- | --- | --- | --- |
| Prossen | 5.69 | 0.97 | 179.2 | 43.9 | 54.2 | 119.7 | 696.4 | 320.6 | 986.9 | 664.8 | 557.9 | 629.2 | 550.2 | 331.6 | 442.7 | 370.7 | 62.1 | 348.5 |
| Meißen. | 5.79 | 0.52 | 199.7 | 212.5 | 140.5 | 156.9 | 708.9 | 429.6 | 1034.7 | 712.2 | 605.9 | 694.4 | 608.1 | 371.4 | 467.5 | 440.4 | 74.1 | 490.2 |
| Barby | 4.78 | 0.44 | 207.2 | 45.0 | 42.5 | 135.5 | 641.6 | 217.6 | 892.0 | 637.4 | 263.8 | 416.8 | 344.0 | 198.1 | 244.6 | 156.2 | 39.2 | 262.0 |
| Cumlosen | 3.94 | 0.31 | 470.1 | 36.8 | 39.9 | 110.7 | 503.9 | 156.4 | 558.0 | 449.2 | 219.2 | 296.8 | 226.4 | 177.5 | 133.5 | 96.4 | 21.8 | 167.5 |
| Neuenfelde | 4.44 | 0.23 | 104.8 | 26.2 | 21.2 | 43.9 | 165.4 | 79.4 | 231.4 | 153.1 | 119.2 | 117.4 | 169.2 | 84.7 | 95.9 | 107.5 | 17.8 | 139.7 |

**Table S5:** C_total_ of seven PCBs in SPM samples from the River Elbe in µg kg^-1^ (dw).

| Station | PCB 28 | PCB 52 | PCB 101 | PCB 118 | PCB 153 | PCB 138 | PCB 180 |
| --- | --- | --- | --- | --- | --- | --- | --- |
| Prossen | 14.11 | 5.74 | 13.59 | 7.81 | 53.44 | 33.65 | 40.70 |
| Barby | 1.91 | 1.31 | 1.78 | 1.75 | 3.23 | 2.63 | 1.36 |
| Cumlosen | 1.47 | 0.89 | 1.53 | 1.72 | 3.91 | 2.85 | 2.44 |
| Neuenfelde | 0.37 | 0.24 | 0.34 | 0.27 | 0.94 | 0.49 | 0.48 |

**Table S6:** C_total_ of PAHs in SPM samples from the River Elbe in µg kg^-1^ (dw).

|  | NAP | ACY | ACE | FLU | PHE | ANT | FLA | PYR | BaA | CHR | BbF | BkF | BaP | InP | DBA | BghiP |
| --- | --- | --- | --- | --- | --- | --- | --- | --- | --- | --- | --- | --- | --- | --- | --- | --- |
| Prossen | 401.5 | 55.8 | 81.7 | 81.5 | 696.0 | 113.8 | 1154.7 | 914.8 | 394.6 | 616.8 | 702.2 | 249.4 | 351.3 | 356.7 | 89.6 | 319.1 |
| Barby | 630.3 | 41.6 | 54.0 | 82.1 | 371.8 | 83.5 | 462.6 | 376.6 | 145.2 | 237.8 | 227.0 | 74.5 | 103.1 | 113.5 | 24.2 | 56.7 |
| Cumlosen | 367.1 | 31.4 | 29.6 | 58.3 | 294.1 | 73.2 | 439.0 | 350.3 | 149.4 | 225.0 | 245.3 | 77.6 | 101.3 | 127.5 | 32.0 | 18.0 |
| Neuenfelde | 21.3 | 2.8 | 3.9 | 6.1 | 30.1 | 8.4 | 44.8 | 37.1 | 18.1 | 30.0 | 32.6 | 11.4 | 16.2 | 17.8 | 4.3 | 8.0 |

**Table S7:** C_free_ of PCBs in sediment samples from the River Elbe in pg L^-1^.

| Station |  | PCB 28 | PCB 52 | PCB 101 | PCB 118 | PCB 153 | PCB 138 | PCB 180 |
| --- | --- | --- | --- | --- | --- | --- | --- | --- |
| Prossen | mean | 68.7 | 51.0 | 19.8 | 3.9 | 14.6 | 15.3 | 4.5 |
|  | SD n=3 | 9.8 | 14.0 | 4.2 | 0.8 | 5.0 | 5.0 | 1.9 |
| Meißen | mean | 0.00 | 133.89 | 0.00 | 1.99 | 12.91 | 17.46 | 5.16 |
|  | SD n=2 | 0.00 | 25.61 | 0.00 | 1.99 | 0.84 | 2.11 | 0.29 |
| Barby | mean | 37.6 | 52.6 | 20.1 | 3.0 | 7.9 | 9.1 | 2.0 |
|  | SD n=4 | 3.9 | 7.7 | 7.7 | 0.2 | 0.3 | 0.2 | 0.1 |
| Cumlosen | mean | 44.0 | 81.7 | 0.0 | 5.4 | 16.9 | 19.0 | 6.7 |
|  | SD n=2 | 62.3 | 22.9 | 0.0 | 0.3 | 0.3 | 1.7 | 0.1 |
| Neuenfelde | mean | 12.57 | 19.01 | 12.27 | 2.32 | 4.60 | 4.82 | 0.71 |
|  | SD n=3 | 1.22 | 1.02 | 2.08 | 0.37 | 0.90 | 0.89 | 0.13 |

**Table S8:** C_free_ of PAHs in sediment samples from the River Elbe in pg L^-1^.

| Station |  | Fl | Phen | Anth | Fluo | Pyr | BaA | Chr | BbF | BkF | BaP | InP | DBA | BghIP |
| --- | --- | --- | --- | --- | --- | --- | --- | --- | --- | --- | --- | --- | --- | --- |
| Prossen | mean | 26205.9 | 27260.1 | 6461.2 | 8682.6 | 5227.3 | 312.4 | 471.8 | 146.1 | 43.4 | 256.8 | 8.7 | 10.6 | 1.9 |
|  | SD n=3 | 5627.6 | 6401.4 | 1240.8 | 1197.1 | 673.2 | 49.4 | 51.1 | 29.8 | 5.2 | 181.8 | 1.5 | 4.5 | 0.1 |
| Meißen | MW | 46183.9 | 42354.2 | 7111.0 | 866.4 | 9753.0 | 567.5 | 766.0 | 0.0 | 0.0 | 0.0 | 0.0 | 0.0 | 0.0 |
|  | SD n=2 | 1388.8 | 1507.4 | 1492.1 | 119.9 | 1135.0 | 121.0 | 102.8 | 0.0 | 0.0 | 0.0 | 0.0 | 0.0 | 0.0 |
| Barby | MW | 11874.0 | 11330.8 | 1122.6 | 2464.6 | 1792.5 | 118.1 | 151.1 | 105.1 | 62.1 | 0.0 | 4.0 | 0.0 | 1.2 |
|  | SD n=4 | 1210.5 | 1046.5 | 128.7 | 133.9 | 83.6 | 12.6 | 8.4 | 27.4 | 12.4 | 0.0 | 0.8 | 0.0 | 0.1 |
| Cumlosen | MW | 18114.1 | 17096.0 | 2742.0 | 3299.6 | 2545.1 | 168.2 | 225.7 | 0.0 | 0.0 | 0.0 | 0.0 | 0.0 | 0.0 |
|  | SD n=2 | 196.0 | 4133.8 | 360.4 | 359.0 | 191.2 | 11.0 | 5.7 | 0.0 | 0.0 | 0.0 | 0.0 | 0.0 | 0.0 |
| Neuenfelde | MW | 9032.2 | 11115.3 | 953.5 | 1720.2 | 1409.7 | 56.9 | 135.3 | 55.9 | 16.1 | 106.1 | 3.7 | 0.0 | 0.9 |
|  | SD n=3 | 2270.7 | 531.0 | 98.8 | 115.5 | 52.0 | 2.9 | 2.6 | 14.7 | 2.3 | 11.1 | 1.1 | 0.0 | 0.2 |

**Table S9:** C_free_ of PCBs in SPM samples from the River Elbe in pg L^-1^.

| Station |  | PCB 28 | PCB 52 | PCB 101 | PCB 118 | PCB 153 | PCB 138 | PCB 180 |
| --- | --- | --- | --- | --- | --- | --- | --- | --- |
| Prossen | MW | 80.2 | 86.8 | 34.4 | 6.7 | 26.4 | 27.0 | 8.9 |
|  | SD n=6 | 9.4 | 15.6 | 7.4 | 0.5 | 2.0 | 1.7 | 0.8 |
| Zehren (Meißen) | MW | 51.7 | 112.2 | 34.7 | 8.2 | 26.6 | 26.9 | 8.8 |
|  | SD n=4 | 7.4 | 33.4 | 4.0 | 0.6 | 3.3 | 3.5 | 1.0 |
| Barby | MW | 66.8 | 151.4 | 20.3 | 5.1 | 13.1 | 15.7 | 4.1 |
|  | SD n=4 | 4.6 | 39.7 | 8.4 | 0.1 | 0.6 | 0.8 | 0.5 |
| Cumlosen | MW | 36.3 | 50.0 | 15.0 | 3.2 | 7.7 | 8.7 | 2.2 |
|  | SD n=4 | 10.8 | 21.4 | 6.8 | 0.7 | 1.2 | 0.8 | 0.1 |
| Neuenfelde | MW | 35.3 | 72.7 | 31.5 | 4.3 | 16.1 | 15.1 | 3.5 |
|  | SD n=4 | 4.3 | 7.8 | 1.5 | 0.4 | 0.3 | 0.3 | 0.1 |

**Table S10:** C_free_ of PAHs in SPM samples from the River Elbe in pg L^-1^.

|  |  | Phen | Anth | Fluo | Pyr | BaA | Chr | BbF | BkF | BaP | InP | BghIP |
| --- | --- | --- | --- | --- | --- | --- | --- | --- | --- | --- | --- | --- |
| Prossen | MW | 18200.6 | 2385.3 | 6932.7 | 4567.1 | 269.8 | 410.9 | 186.1 | 97.3 | 246.4 | 11.4 | 2.2 |
|  | SD n=6 | 2435.7 | 244.1 | 392.4 | 287.7 | 31.2 | 42.0 | 61.6 | 43.5 | 22.8 | 3.5 | 0.4 |
| Zehren (Meißen) | MW | 13915.4 | 1424.3 | 4286.6 | 3058.3 | 155.6 | 312.3 | 244.3 | 142.2 | 219.8 | 12.5 | 2.2 |
|  | SD n=4 | 2056.9 | 160.9 | 525.3 | 317.0 | 33.7 | 61.9 | 84.6 | 49.6 | 43.4 | 4.3 | 0.9 |
| Barby | MW | 16224.4 | 1486.5 | 2959.9 | 2235.0 | 137.0 | 204.2 | 161.1 | 41.4 | 570.9 | 10.1 | 1.9 |
|  | SD n=4 | 981.5 | 582.5 | 294.1 | 96.5 | 6.0 | 16.2 | 23.1 | 15.9 | 110.9 | 2.5 | 0.2 |
| Cumlosen | MW | 15108.8 | 2159.5 | 3910.4 | 2740.0 | 142.7 | 213.8 | 98.9 | 31.1 | 97.5 | 6.0 | 1.1 |
|  | SD n=4 | 4772.1 | 409.6 | 158.9 | 121.6 | 12.7 | 24.9 | 18.2 | 5.8 | 82.3 | 3.9 | 0.8 |
| Neuenfelde | MW | 14197.5 | 2006.0 | 2984.6 | 2293.5 | 115.7 | 202.8 | 79.7 | 34.4 | 192.0 | 7.7 | 1.6 |
|  | SD n=4 | 1011.7 | 865.7 | 109.5 | 118.2 | 6.9 | 6.3 | 1.0 | 1.4 | 33.7 | 1.1 | 0.1 |

FLIEDNER, A., RÜDEL, H., LOHMANN, N., BUCHMEIER, G. & KOSCHORRECK, J. 2018. Biota monitoring under the Water Framework Directive: On tissue choice and fish species selection. *Environmental Pollution,* 235**,** 129-140.
